# Psychosocial needs of patients and spouses justify a position of psychosocial health professionals in the multidisciplinary care for Parkinson's disease

**DOI:** 10.1016/j.prdoa.2020.100064

**Published:** 2020-07-03

**Authors:** Annelien Duits, Colin van der Heijden, Masja van het Hoofd, Gabriel Roodbol, Mark Tiemessen, Marten Munneke, Maxime Steppe

**Affiliations:** a Department of Medical Psychology, Maastricht University Medical Centre (MUMC)+, P.O. Box 5800, 6202, AZ, Maastricht, the Netherlands; b Department of Psychiatry and Neuropsychology, School for Mental Health and Neuroscience, Maastricht University, PO Box 616, 6200, MD, Maastricht, the Netherlands; c ParkinsonNet, RadboudUMC, Reinier Postlaan 4, 6525, GC, Nijmegen, the Netherlands; d Department of Medical Psychology, RadboudUMC, PO box 9101, HB, Nijmegen, the Netherlands; e Parkinson Vereniging [Dutch Parkinson's Disease Association], PO Box 46, 3980 CA Bunnik, the Netherlands; f Department of Psychiatry, RadboudUMC, PO Box 9101,6500, HB, Nijmegen, the Netherlands

## Abstract

**Introduction:**

Living a life with Parkinson's Disease (PD) is a challenge for both patients and spouses. Patients have to cope with an increasing limitation in all domains of their daily life and spouses need to adjust to these changes. The focus of this study is on exploring, both quantitatively and qualitatively, the psychosocial needs of both patients with PD and spouses.

**Methods:**

An online survey with 11 themes, related to dealing with a chronic disease, was sent by an email to patients and spouses and two focus groups were planned, one with patients and one with spouses. Data from the survey were quantitatively analysed and audiotapes from the focus groups were transcribed verbatim and combined with notes.

**Results:**

Percentages of relevance are higher than 50% for all the themes, whereas those of the need for and received support are all lower than 50%. Focus groups revealed a negative image of psychosocial therapy and associations with failure, but also difficulties in signalling problems by professionals, little attention for spouses and limited access to specialized psychosocial care.

**Conclusion:**

Based on this exploration, there appears to be a threshold to ask for psychosocial support on the one hand and to find the right professional on the other hand. A permanent position for psychosocial health professionals in the multidisciplinary Parkinson teams and networks may close the gap between ‘supply and demand’.

## Highlights

• Living a life with Parkinson's Disease (PD) is a challenge for both patients and spouses
• Psychosocial therapy has a negative image leading to a high threshold to ask for support.
• The access to psychosocial professionals with expertise in PD is limited
• Psychosocial professions should have a permanent position in the multidisciplinary teams and networks to normalize their image and close the gap between needs and support.

## Introduction

Parkinson's disease (PD) is a complex neurodegenerative disorder, which has an important psychosocial impact on the lives of patients and their spouses. During the disease course, patients experience progressive motor and non-motor symptoms, the latter including neuropsychiatric symptoms such as anxiety, depression and cognitive impairment. Patients have to cope with an increasing limitation in all domains of their daily life. Also spouses need to adjust to these changes and may feel uncertain about their ability to take care of a person with PD [1]. Knowledge on how patients with PD and their spouses and/or family carers cope with the challenges of an unpredictable body, fear of falling or any of the other consequences of the disease may improve individualized care and rehabilitation [2].

Both multi-disciplinary and personalized treatment for PD has increased in recent years, taking into account the complexity of the disease and acknowledging the psychosocial challenges. ParkinsonNet, founded in 2004 as a network of specialized physical therapists, consists to date of 71 regional networks around the Netherlands, and is expanding internationally [3]. The present Dutch network includes professionals in different disciplines, all specifically trained in treating patients with PD [4]. Although the psychosocial impact of the disease is high, the participation of psychosocial professionals in the network is relatively low (i.e. 5% against 38% for physical therapists). The current study quantitatively and qualitatively explored the psychosocial needs of both patients with PD and spouses to answer the question whether there is a demand for psychosocial professionals to support dealing with the disease.

## Methods

### Participants

Those eligible to participate were patients with PD and spouses willing to complete an online survey and discuss their personal situation in a focus group. Recruitment was organized by the Dutch Parkinson's Disease Association. Two focus groups were formed, one including patients and the other one including spouses. These groups were led by a psychologist and a nurse practioner mental health (patients) and a social worker (spouses). Written informed consent was obtained prior to participation.

### Procedures

An online survey was sent by a link in a (digital) newsletter to all the members of the Dutch Parkinson's Disease Association (MvH), both patients and their spouses (a total of 5688 emailadresses). The survey included 11 psychosocial themes (see Table 1) derived from Pool and colleagues [5]. Participants were asked to rate the importance of the theme and whether they needed support to deal with it. If they had any support they were asked from whom they received the support, including psychosocial health care professionals.

**Table 1:** Themes online survey patients: relevance, need for and received support.

	Patients	(*n* = 1073)			Spouses	(*n* = 353)		
Themes	Relevant (extremely relevant)	Need for support	Received support	Psycho-social support^a^	Relevant (extremely relevant)	Need for support	Received support	Psycho-social support^a^
1) Recognize body signals and take these into account	81,8 (33,8)	36,7	36,8	29,9	99,4 (58,1)	34,8	30,4	33,9
2) Ratio rest and activity	80,6 (30,4)	33,2	29,6	25,5	99,7 (51,6)	34,0	24,4	30,5
3)Fitting rules and therapies into daily life	85,6 (40,7)	31,2	38,9	16,2	99,7 (57,5)	28,9	23,9	14,5
4) Dealing with losses	80,6 (35,3)	38,6	28,4	36,8	100 (54,7)	42,2	21	31,5
5) Dealing with uncertainty and feelings of shame and guilt	53,8 (18,8)	24,5	19,3	39,2	84,1 (30,6)	23,6	15,9	40,0
6) Adjusting personal interests and ambitions	73,1 (26,0)	31,2	21,1	30,9	76,2 (28,3)	18,0	18,1	17,4
7) Dealing with incomprehension (due to the environment)	48,9 (15,5)	18,6	13,4	30,4	85 (28,9)	17,7	12,5	34,8
8) Maintaining social contacts	69,6 (33,3)	23,9	13,7	24,6	93,8 (40,5)	22,0	7,6	42,3
9) Intimate relationships and sexuality	56,3 (17,7)	17,9	8	30,5	79,3 (18,1)	16,5	28,3	14,4
10) Dealing with health care contacts	69,8 (25,0)	25,1	18,5	19,4	96,6 (43,3)	31,9	31,2	19,4
11) Maintaining in the work situation*	69,2 (9,9)	27,5	16,8	21,5				
11) Finding the way in health care					97,2 (50,4)	49,7	0	0

All data are percentages (%) of the total sample of patients and spouses except for ^a^ = % of those who received support with ‘psychosocial’ referring to psychiatrists, psychologists and social workers; *n = 306.

Additionally, two focus groups were planned in parallel. Eighteen patients were randomly selected to participate in the patient focus group, based on a positive response to the request (i.e., last question in the online survey). Their spouses were invited to participate in a separate spouse focus group. The moderators (AD, CvH) used a semi-structured topic guide based on the four most relevant themes from the survey, and psychosocial support in general (questions about the participants' experience with need for and received support). The interviews were audiotaped. Two researchers (MS, GR) took additional notes, to ensure identification of potentially relevant non-verbal information or cues presented by the participants. Each meeting had a duration of approximately 90 min.

### Data analysis

Data from the survey were analysed by SPSS (version 25) using descriptive analyses (means and frequencies) and chi-square analyses, comparing patients and spouses, and men and women on the relevance of themes. Audiotapes from the focus groups were transcribed verbatim (MS, CvH) and combined with notes. Transcripts were analysed independently by authors CvdH and MS using the qualitative data analysis and research software Atlas Ti. 8.4.15. The texts were read thoroughly and open codes were applied to all aspects of the content. Interpretation and integration of categories into themes was discussed and finalized with all authors. Quotations were selected based on representativeness by MS, and all authors checked their relevance.

## Results

### Participants

The response rate of the online survey was 25% (1426 / 5688 members). A total of 1073 PD patients, 625 men and 448 women, completed the survey with a mean age of 67,6 years (sd 8,2; range 31–88). Almost half of the patients (49,4%) had a disease duration between 0 and 5 years, 29,7% between 6 and 10 years, 13,3% between 11 and 15 years, 5.1% between 16 and 20 years and 2.4% longer than 20 years. Only 3 patients lived in a nursing home. More than half of the patients (50,2%) had a high education level (>18 years of education) and 28,5% were working full- or part-time.

A total of 353 spouses, 112 men and 241 women, completed the online survey with a mean age of 68,7 years (sd 7,7; range 39–86) and with 34,8% of them having a high education level (> 18 years of education).

Twelve of 18 patients (8 men and 4 women; mean age 68,8 years; range 54–73) and 6 of their spouses (3 men and 3 women; mean age 72 years, range 65–86) were available to participate in the focusgroups. Half (6) of the patients had a disease duration between 0 and 5 years, 4 patients between 11 and 15 years, 1 between 16 and 20 and 1 longer than 20 years.

### Response on the survey patients and spouses

See Table 1 for the respective themes (1−11), their relevance (percentages of patients and spouses indicating that this theme was relevant/extremely relevant for them) and the percentages of both the need for and received support for both the patients and the spouses. Based on chi-square analyses, the percentages of relevance for spouses (range 76,2–100) are significantly higher than those for patients (range 48,9-85,6), for all themes (p 〈0,00). When comparing men and women, either patient or spouse, the percentages of relevance related to maintaining social contacts and intimate relationships (theme 8 and 9) are higher for women than for men (*p* < .01). The percentages of need for support vary between 17,9–38,6% for patients and between 16,5-49,7% for spouses. The percentages of received psychosocial support are highest (40%) in dealing with uncertainty and guilt (theme 5) for patients and in maintaining social contacts for (theme 8) spouses (42%).

### Focus groups

Two main topics were identified for the patients: 1) high threshold to ask for support and 2) when in need of help it is difficult to find a psychosocial professional. The first topic is related to feelings of failure, giving up and admitting that one is not able to deal with a problem. Furthermore, there is uncertainty about what psychosocial support entails. Finally, there are already many professionals involved in the overall health care for PD. The second topic is related to the problem of recognizing and signalling psychosocial problems and referring patients to the right person. See Table 2 for some related quotes.

**Table 2:** Main topics and illustrative transcripts from the focus groups with patients (*n* = 12) and spouses (*n* = 6).

**Patients: Topic 1 high threshold to ask for psychosocial support**
“You are afraid to talk about emotions that you wait for a long time and think long about yes I would do that now because that again gives a certain dependence on such a person”.
“If you are a person who does not want to be dependent, absolutely does not want to be dependent, then you have a problem if you have to ask for help at a given moment”.
“You are not sure, when I go to physiotherapy I know exactly what happens and I can take a somewhat distant attitude. I say ok I do my balance exercises and I do my running exercises and then it is finished, tonight, again tonight for example. But when I go to the psychologist, yes, I am not quite sure what happens in that hour”.
“I want to talk about it but don't know if I need the whole project of going into therapy”.
“If I am with my psychologist then I slam shut and I think oh, well we are going to tackle a problem, oh dear.”
“Yes, social work, that's a bit more ordinary, isn't it? That is closer to home. That also sounds to practice I think and people think that is a bit easier I think”.
“Yes, that a number of people around me could handle this very well and also knew a lot so I wouldn't want to have someone else with me”.
**Patients: Topic 2 high threshold to find psychosocial support**
“A whole lot of Parkinson's patients are depressed and, to a greater or lesser degree, anxious. And there I think, is my idea anyway, I see that there is a huge group that gets no help. Because they don't ask for it”.
“With the daily information that professionals in the ParkinsonNet also have, why not signalling that this specific gentleman could benefit from that or that psychotherapy”.
“Yes with me it was the case that the physiotherapist was in the network, also occasionally said you might go and talk with someone. But when I asked where and whom? Then she didn't really know”.
**Spouses: Topic 1 little attention for spouses and caregivers**
“What we often encounter is the fact that you are not seen, even though you are with the Parkinson's nurse or neurologist, your story is not addressed”.
“To have the feeling that it is not only about your husband or partner who is sick, but that it also applies to you. At least I got a lot out of that case manager dementia in this case someone who says I will see how you are, I notice how your husband is, I see how you react to it - what can I do for you to do? What do you need now”.
“Look, there comes a lot from your own past during that entire process of caregiving. You think that you are strong, and again, that is me and stubbornly so you think that you can do a lot yourself but then you still come to a limit that you feel that you are doing it but that it is actually no longer possible”.
“It only comes, ‘How is your partner?’ What does he bump into? Does he still need medication?” Things like that. And sometimes people say “How are you?” But you don't actually give a decent answer to that. Because the focus is on the partner” .
**Spouses: Topic 2 lack of knowledge related to the consequences of Parkinson's Disease**
“I think there is not enough knowledge about Parkinson's. Also with our general practitioner (GP), maybe with other doctors again I do not know that. But with our GP, yes he doesn't actually know either.”
“So, you have to find out a physiotherapist or an occupational therapist or speech therapist who uses ParkinsonNet because they get a course, they have that specialization Parkinson. But now someone else for us, I think. You know, someone who knows a lot about Parkinson's. And that's it, a social worker can give me a lot of things, but they don't know anything about Parkinson's. And such a person should, for example, also be in such a network.”
“Look at the municipality, they send a civil servant, you talk to him and they know that they all know nothing about it because they come for all sorts of other things too. Ultimately, they can only refer to the insurers”.

Also two main topics were identified for the spouses: 1) too little attention for the spouses (the focus is mostly on the patient) and 2) a lack of understanding and empathy in general health care and the municipalities, who assess whether care support is indicated in the home situation. Although spouses may experience a (personal) threshold to ask for support, their main problem is to find the right help with knowledge of PD. See Table 2 for some related quotes.

## Discussion

The present study explored the psychosocial needs of patients with PD and their spouses, both quantitatively by an online survey and qualitatively by focus groups. The online survey included 11 themes related to dealing with a chronic disease, with spouses showing higher percentages of relevance than patients. Women showed higher percentages of relevance than men for the items related to maintain social contact and intimate relationships. Overall, percentages of relevance appeared relatively high, whereas those of the need for and perceived support are low to moderate suggesting that relevance does not necessarily lead to (need of) support. This discrepancy is in line with findings in other chronic ill populations, indicating that high psychological distress levels are not by definition related to a need for psychosocial support [6,7].

Based on the information from the focus groups the need for support might be masked since patients experience a high threshold to ask for psychosocial support. The threshold seems to be raised because of the stigma that therapy is for the weak and the uncertainty about the content of the interventions. Furthermore, patients may feel burdened by too many involved professionals. Spouses may also experience a high personal threshold to ask for help but they mostly feel neglected. All the attention is going to the patient. Both patients and spouses indicate that if in need for support it is difficult to find the right person with knowledge of PD.

Psychosocial adaptation to the disease is an ongoing process especially when living with a chronic and progressive disease such as PD [1]. Programs that assist in the use of community resources, increasing personal networks and social support are supposed to be the future direction for the management of neurodegenerative conditions, not only in the late phase of the disease [8]. However, the indication for support in terms of who is taking care of it and when, is not clear yet. PD specialist nurses can provide a listening ear and practical and emotional support in coping with challenges and can refer to specialized care when there are specific problems. When dealing with the municipalities, issues of acceptance and difficulties with coping, social workers are helpful and in case of neuropsychiatric symptoms psychologist and psychiatrists can be consulted. Whereas psychological and psychiatric treatment is usually symptomatic, psychosocial support aims to help in developing emotional and cognitive strategies to live well despite of PD (e.g., self-management interventions).

So far, the availability of psychosocial professionals with specific knowledge about PD is limited with only small percentages of social workers and psychologists in the Dutch ParkinsonNet networks. By reducing the threshold of patients to ask for support, the potential case-load for psychosocial professionals may increase, which is helpful to enter the networks. Education of patients and spouses (or family carers) with information related to the challenge of adjusting to the disease may be helpful to close the gap between ‘supply and demand’. In collaboration with the Dutch Parkinson's Disease Association, we developed a flyer to share with all members. This flyer, including the 11 themes from the online survey, is designed to be a guideline for self-monitoring. Self-monitoring is considered an important skill to manage daily life leading to self-awareness. As such, it can form the basis for self-care activities and for consultation with healthcare professionals. Self-monitoring is one component in the broader concept of self-management [9]. In collaboration with the Dutch ParkinsonNet, we also developed an animation and checklist for professionals to signal psychosocial problems. So far, there are no data yet related to the impact of both actions in terms of unmasking the true need of support (e.g., increasing numbers of referrals and psychosocial professionals in the network).

This study is not without limitations. First, we purposefully selected a sample to represent a wide range of personal and disease-related characteristics, but it is likely that those patients who are most seriously affected were not represented. As a consequence, generalizability of the results for spouses may be limited as well. Second, the relevance of psychosocial themes is probably not limited to PD and the comparison with non-PD patients and their spouses would be of additional value to substantiate our findings. Furthermore, comparing PD and non-PD patients may improve our understanding of the gender differences and differences between patients and spouses, whether these are specific for PD or not. Third, and also related to the design of the study, we used only two focus groups, whereas research suggests that most issues around a research topic will be captured after the fourth group [10]. Finally, the present data reflect the Dutch situation, while issues as stigma and the organisation of health care may vary in different cultures. Although the present findings require further validation, they justify a position of psychosocial health professionals in the overall management of PD.
